# Development of a Portable Rapid Detection Method for Porcine Epidemic Diarrhea Virus Using Reverse Transcription-Recombinase-Aided Amplification Technology

**DOI:** 10.3390/ani15020281

**Published:** 2025-01-20

**Authors:** Yiran Zhao, Weijie Yi, Qicheng Yang, Jiahao Li, Yanke Shan, Fei Liu

**Affiliations:** 1 Joint International Research Laboratory of Animal Health and Food Safety of Ministry of Education, Single Molecule Biochemistry & Biomedicine Laboratory (Sinmolab), College of Veterinary Medicine, Nanjing Agricultural University, Nanjing 210095, China; zhaoyr1998@163.com (Y.Z.); 15060029212@163.com (W.Y.); kikuchanj_0618@163.com (Q.Y.); feiliu24@njau.edu.cn (F.L.); 2 Key Laboratory of Veterinary Biological Engineering and Technology, Ministry of Agriculture, Institute of Veterinary Medicine, Jiangsu Academy of Agricultural Sciences, Nanjing 210014, China; ljh_jaas@126.com

**Keywords:** porcine epidemic diarrhea virus, isothermal amplification technology, reverse transcription-recombinase-aided amplification, point-of-care detection

## Abstract

Porcine epidemic diarrhea virus (PEDV) continues to spread globally, causing clinical symptoms in piglets, including watery diarrhea, vomiting, and dehydration. Its exceptionally high morbidity and mortality rate contributes significantly to the economic losses of the swine industry. The continuous genetic mutations of PEDV have compromised the effectiveness of classical strain vaccines. Early detection and accurate diagnosis are therefore crucial for controlling its further spread. Developing a detection method that is user-friendly, highly sensitive, and efficient is crucial for disease control. In this study, a point-of-care rapid detection method for PEDV was successfully established using reverse transcription-recombinase-aided amplification (RT-RAA) technology. This method enables results to be obtained within 20 min of amplification at a constant temperature of 42 °C. It demonstrates high sensitivity, with a detection limit as low as 1 copy/μL, and shows strong specificity, with no cross-reactivity observed with seven other common swine pathogens. When applied to clinical samples, the results were 100% consistent with those obtained by RT-qPCR. This method is distinguished by its portable instrumentation and simple operation, making it particularly suitable for resource-constrained settings.

## Simple Summary

Porcine epidemic diarrhea virus (PEDV), a member of the Coronaviridae family, is responsible for severe watery diarrhea, vomiting, and dehydration, leading to significant mortality in neonatal piglets. A rapid detection system for PEDV, based on reverse transcription-recombinase-aided amplification (RT-RAA) technology, was successfully developed in this study. The developed detection system is characterized by its simplicity in operation, high sensitivity, and stability. Additionally, when integrated with a compact blue-light detection device, it facilitates on-site visual detection, making it suitable for field applications.

## 1. Introduction

Porcine epidemic diarrhea (PED) is an acute, highly contagious intestinal disease caused by porcine epidemic diarrhea virus (PEDV). PED induces clinical signs, including vomiting, diarrhea, and dehydration in piglets, with mortality reaching up to 100% in neonatal piglets [1]. PEDV belongs to the genus Alphacoronavirus of the family Coronaviridae within the order Nidovirales. It is an enveloped, single-stranded, positive-sense RNA virus with a diameter of 95–190 nm [2]. The PEDV genome is approximately 28 kb in length and encodes four structural proteins—spike (S), envelope (E), membrane (M), and nucleocapsid (N) proteins—sixteen nonstructural proteins (nsp1–nsp16), and an accessory protein ORF3 [3]. PEDV was first identified in the United Kingdom in 1971, after which it rapidly spread worldwide [4]. In 1973, an outbreak of acute diarrhea resembling transmissible gastroenteritis (TGE) was observed in Shanghai, China, but it was not until 1984 that PEDV was confirmed as the causative agent [5]. In October 2010, a highly virulent variant strain of PEDV emerged in China, characterized by high morbidity rates (nearly 100%) and high mortality rates (80–100%) in neonatal piglets [6]. Sequence insertions and mutations found in this variant strain may have conferred enhanced pathogenicity, compromising the efficacy of existing vaccines. This ultimately led to severe diarrhea outbreaks in pig farms in China in 2011 [7]. Currently, this highly pathogenic PEDV strain has spread extensively across many countries in Europe, Asia, and the Americas [8,9,10], causing substantial economic losses to the global swine industry [11]. Due to the ongoing genetic mutations of PEDV, classical vaccines have become less effective in preventing and controlling infections caused by variant strains [12]. Early detection and accurate diagnosis of PEDV are critical to controlling its spread in pig farms. Consequently, the development of a simple, sensitive, accurate, and rapid point-of-care detection method for PEDV is significant for disease prevention and control.

Various methods have been developed for detecting PEDV, including virus isolation, immunofluorescence assay (IFA), immunohistochemistry (IHC), polymerase chain reaction (PCR), and enzyme-linked immunosorbent assay (ELISA) [13,14,15,16,17]. Among these, molecular techniques such as reverse transcription-quantitative PCR (RT-qPCR) have become the preferred approach for diagnosing PEDV infections due to their sensitivity, specificity, and precision in detecting viral nucleic acids in clinical samples [18]. However, these methods rely on bulky, expensive equipment and involve complex procedures, making them unsuitable for rapid on-site detection, thus limiting their widespread application [19]. Additionally, with technological advancements, emerging diagnostic methods, such as the CRISPR-Cas system and microfluidic chip technologies, have been introduced. These methods provide significant improvements in sensitivity and accuracy, enabling high-performance detection. However, their reliance on specialized equipment, complex operational requirements, and high costs still restricts their practical application in field settings or resource-limited environments [20,21]. This highlights the need for novel technologies to address these challenges. Isothermal amplification technology (IAT) has emerged as a novel nucleic acid amplification method in recent years. It maintains a constant temperature throughout the reaction process, enabling rapid nucleic acid amplification by adding enzymes with distinct activities and specific substrates [22]. Compared to traditional PCR, IAT is faster, more efficient, and does not require complex thermal cycling equipment [23]. Among IAT methods, loop-mediated isothermal amplification (LAMP) and recombinase-aided amplification (RAA) are widely used. However, LAMP faces several challenges, including complex primer design, susceptibility to aerosol contamination, and the potential to produce false positives due to specificity issues [24]. In contrast, RAA offers simpler primer design, rapid reactions, and the ability to amplify nucleic acids at relatively low temperatures (37–45 °C) with high specificity and efficiency, making it particularly advantageous for on-site detection [25].

**Figure 1:**
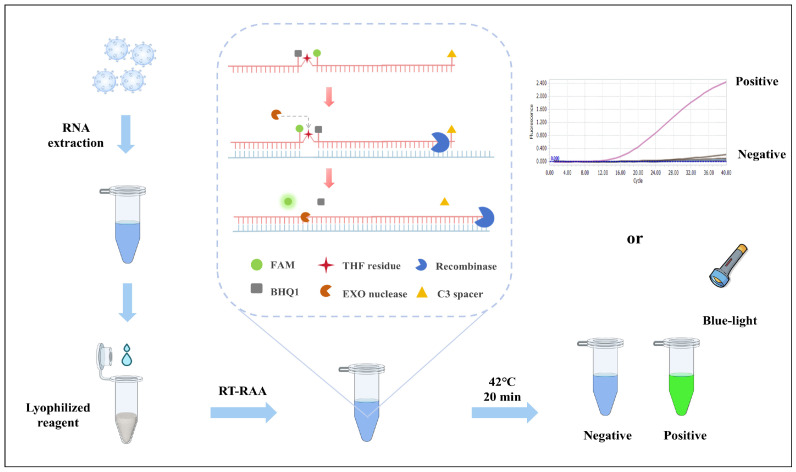
Schematic diagram of the principle of RT-RAA technology for detecting PEDV.

This study successfully developed a point-of-care detection method for PEDV based on reverse transcription-recombinase-aided amplification (RT-RAA) technology, which integrates visual and real-time fluorescence detection (Figure 1). The method operates at a constant temperature of 42 °C and provides results within 20 min. It exhibits high sensitivity, with a minimum detection limit of 1 copy/μL. The assay demonstrates outstanding specificity, showing no cross-reactivity with porcine reproductive and respiratory syndrome virus (PRRSV), classical swine fever virus (CSFV), pseudorabies virus (PRV), porcine circovirus type 2 (PCV2), porcine deltacoronavirus (PDCoV), porcine rotavirus (PoRV), or transmissible gastroenteritis virus (TGEV). When applied to clinical samples, the results were 100% consistent with those obtained by RT-qPCR. In summary, this detection method offers high sensitivity, excellent specificity, and ease of operation without requiring large, sophisticated equipment. It is well-suited for on-site detection and demonstrates significant potential for practical applications in pig farms and other field settings.

## 2. Materials and Methods

### 2.1. Reagents and Viruses

The RNA isothermal rapid amplification kit (fluorescent type) was purchased from Amplification Future Biotechnology Co., Ltd. (Changzhou, China). The porcine epidemic diarrhea virus (PEDV) detection kit (fluorescent PCR method) was purchased from Shanghai Xunya Biotechnology Co., Ltd. (Shanghai, China). The virus DNA/RNA extraction kit was purchased from Trans Biotech Co., Ltd. (Beijing, China). The PEDV N gene positive standard plasmid was synthesized by General Bio Co., Ltd. (Chuzhou, China). The CSFV live vaccine and PRV live vaccine were purchased from China Animal Husbandry Industry Co., Ltd. (Beijing, China). Positive samples for TGEV, PRRSV, PDCoV, PoRV, and PCV2 are stored in our laboratory. The clinical samples used in this study comprised fecal and rectal swab samples from pigs with diarrhea, collected across various regions of Liaoning Province between June 2022 and June 2023, including Shenyang, Fuxin, Jinzhou, Chaoyang, and Huludao. All samples were stored at −80 °C until they were needed for further analysis.

### 2.2. Primer and Probe Design and Synthesis

The N gene sequence of the PEDV epidemic strain was retrieved from the GenBank database and analyzed using DNAMAN 9.0 software. Conserved regions identified through the analysis were selected as detection targets. Following the design principles for RAA primers and probes, a series of primers and probes were designed using Primer Premier 5.0 and SnapGene 4.6.4 Viewer software (Table 1), and were synthesized by Sangon Biotech Co., Ltd. (Shanghai, China).

### 2.3. Virus Nucleic Acid Extraction

Following the instructions provided with the DNA/RNA extraction kit, nucleic acids were extracted from CSFV live vaccine, PRV live vaccine, TGEV, PRRSV, PDCoV, PoRV, PCV2 samples, and PEDV clinical samples. The extracted nucleic acids were eluted in 40 μL of DNase/RNase-free water and stored at −80 °C for future use.

### 2.4. Establishment of the PEDV RT-RAA Reaction System

The reaction system was established according to the instructions provided with the RNA isothermal rapid amplification kit (Fluorescent type). The single-tube reaction system had a total volume of 25 μL, consisting of 14.7 μL of A Buffer, 1 μL of 10 μM forward primer (F), 1 μL of 10 μM reverse primer (R), 0.3 μL of 10 μM probe, 2 μL of nucleic acid template, 4.75 μL of DNase/RNase-Free Water, and 1.25 μL of B Buffer, all of which were thoroughly mixed. Since B Buffer serves as the initiator for the amplification reaction, it should be added to the inner surface of the tube lid. After securely closing the lid, the contents were mixed thoroughly. After mixing, the tube was briefly centrifuged to ensure the contents were at the bottom, and then immediately placed into the Roche LightCycler 96 Real-Time PCR System (Roche Diagnostics, Mannheim, Germany). The fluorescence program was set as follows: a constant temperature of 42 °C and a reaction time of 20 min. The results were determined by the intensity of the fluorescence signal. If fluorescence is detected in the reaction tube, it indicates the presence of the target nucleic acid. If no fluorescence is observed, the result is considered negative, indicating that no target nucleic acid was detected. In addition, after amplification, the result can also be determined by blue light illumination. Under blue light excitation, positive samples will show a distinct fluorescence signal, while negative samples will not display significant fluorescence signals.

### 2.5. Screening of RT-RAA Primers

Using the PEDV N gene plasmid as a template, three sets of primer–probe pairs were evaluated for their detection efficiency. Using the PEDV N gene plasmid as a template, three sets of primer–probe pairs were evaluated for their detection efficiency in the PEDV RT-RAA reaction system. The amplification results were verified using agarose gel electrophoresis and fluorescence detection during the reaction process. The earliest amplification onset time and fluorescence signal intensity were assessed using the Roche LightCycler 96 Real-Time PCR System (Roche Diagnostics, Mannheim, Germany). to identify the optimal primer–probe pairs.

### 2.6. Optimization of RT-RAA Reaction Conditions

After the optimal primer–probe combination was determined, the reaction conditions for the PEDV RT-RAA reaction system were optimized using a plasmid containing the PEDV N gene as the amplification template, with only one variable controlled during the process. The optimized reaction conditions were as follows: primer concentrations (0.4, 0.3, 0.2, and 0.1 μM), probe concentrations (0.16, 0.14, 0.12, 0.10, 0.08, and 0.06 μM), reaction temperatures (40, 41, 42, and 43 °C), and reaction time.

### 2.7. Specificity Validation of the PEDV RT-RAA Reaction System

To assess the specificity of the PEDV RT-RAA reaction system, the optimized reaction system was employed to detect nucleic acids extracted from positive samples of PRRSV, CSFV, PRV, PCV2, PDCoV, PoRV, TGEV, and PEDV.

### 2.8. Sensitivity Validation of the PEDV RT-RAA Reaction System

The sensitivity of the PEDV RT-RAA reaction system was assessed by using the optimized reaction system to detect PEDV N gene plasmid standards ranging from 10^6^ copies/μL to 1 copy/μL, with DNase/RNase-free water used as the negative control.

### 2.9. Reproducibility Validation of the PEDV RT-RAA Reaction System

Three plasmid standard samples at different concentrations (10^5^, 10^4^, and 10^3^ copies/μL) were selected as templates to evaluate the reproducibility of the optimized PEDV RT-RAA reaction system. Each sample was tested in triplicate on the first day, followed by repeated testing on the second and third days. Fluorescence signals were detected using a real-time fluorescence quantitative PCR instrument, and the reproducibility of the assay was assessed based on the cycle threshold (CT) values.

### 2.10. PEDV RT-RAA Reaction System Detection of PEDV Clinical Samples

Sixty-three fecal and rectal swab samples from diarrheic pigs were collected, including 27 PEDV-positive and 36 PEDV-negative samples. The optimized PEDV RT-RAA reaction system was employed for detection, and the results were compared with those obtained using RT-qPCR. The clinical applicability of the method was assessed.

## 3. Results

### 3.1. Primer Screening for the PEDV RT-RAA Reaction System

**Figure 2:**
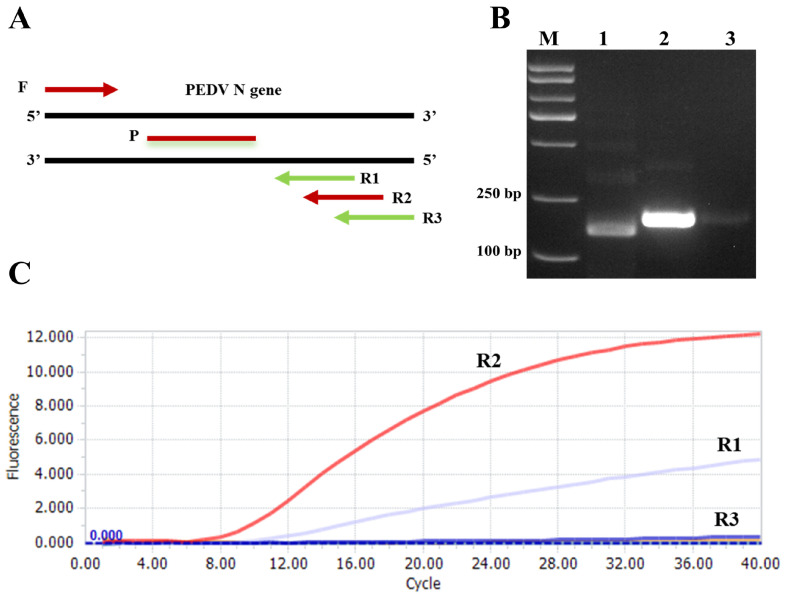
Results of screening for PEDV primers and probes. (**A**) Relative positions of candidate primers and probes within the PEDV detection target. (**B**) The RT-RAA results of the PEDV standard positive plasmid were verified by agarose gel electrophoresis. Numbers 1–3 represent the amplification products produced by primers R1, R2, and R3, respectively. (**C**) The RT-RAA detection results of the PEDV standard positive plasmid were verified by a fluorescence detector.

Amplification efficiency in RAA is influenced by primer combinations. In this study, one upstream primer and three downstream primers (R1, R2, and R3) were designed for the PEDV N gene, and each primer pair was used in RT-RAA (Figure 2A). Amplification products were analyzed using 2% agarose gel electrophoresis. The results demonstrated that the target bands for the three primer pairs were 156 bp, 178 bp, and 189 bp, respectively, which were consistent with the expected sizes shown in Figure 2B, confirming the successful amplification of the target nucleic acids. Fluorescence intensity was further evaluated using real-time quantitative PCR. As shown in Figure 2C, the RT-RAA reaction with downstream primer R2 exhibited the highest fluorescence intensity. Therefore, the combination of upstream primer F and downstream primer R2 was identified as the optimal primer pair.

### 3.2. Optimization of RT-RAA Reaction Conditions

**Figure 3:**
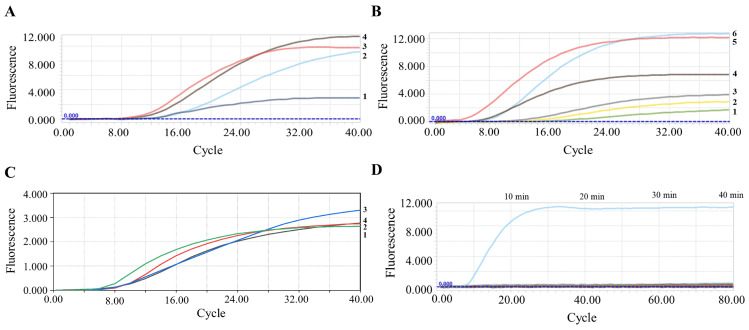
Optimization of reaction conditions for the PEDV RT-RAA reaction system. (**A**) Optimization of primer concentrations in the PEDV RT-RAA reaction system. Numbers 1–4 correspond to 0.1, 0.2, 0.3, and 0.4 μM, respectively. (**B**) Optimization of probe concentrations in the PEDV RT-RAA system. Numbers 1–6 correspond to 0.06, 0.08, 0.10, 0.12, 0.14, and 0.16 μM, respectively. (**C**) Optimization of reaction temperature in the PEDV RT-RAA reaction system. Numbers 1–4 correspond to 40, 41, 42, and 43 °C, respectively. (**D**) Optimization of reaction time in the PEDV RT-RAA system.

After selecting downstream primer R2, further optimization of the RT-RAA reaction conditions was performed. Various combinations of upstream and downstream primer concentrations (0.4, 0.3, 0.2, and 0.1 μM) were tested. RT-RAA detection was performed, and fluorescence intensity was measured using real-time quantitative PCR. As shown in Figure 3A, fluorescence intensity increased progressively with primer concentration and peaked at 0.4 μM. Therefore, the optimal primer concentration was determined to be 0.4 μM.

Next, the probe concentration in the system was optimized by performing RT-RAA reactions with varying concentrations of probes (0.16, 0.14, 0.12, 0.10, 0.08, and 0.06 μM). As shown in Figure 3B, the fluorescence intensity increased progressively with higher probe concentrations, with the highest fluorescence absorbance observed at 0.16 μM. However, a stable fluorescence signal was achieved around the 5th cycle at 0.14 μM, whereas for 0.16 μM, the stable signal appeared only after approximately the 10th cycle. Considering the similar fluorescence absorbance values under the 0.14 μM and 0.16 μM conditions, the optimal probe concentration was ultimately determined to be 0.14 μM.

Then, we optimized the reaction temperature of the RT-RAA system by comparing fluorescence intensities at 40, 41, 42, and 43 °C. The results showed that the strongest fluorescence signal was obtained at 42 °C. Therefore, 42 °C was selected as the optimal reaction temperature for this system (Figure 3C). Finally, we determined the optimal reaction time by comparing the fluorescence intensity changes at different time points. The results showed that the fluorescence curve plateaued after 20 min, with the fluorescence intensity remaining nearly unchanged. Thus, we chose 20 min as the optimal reaction time for this system (Figure 3D).

### 3.3. Specificity Evaluation of the PEDV RT-RAA Reaction System

**Figure 4:**
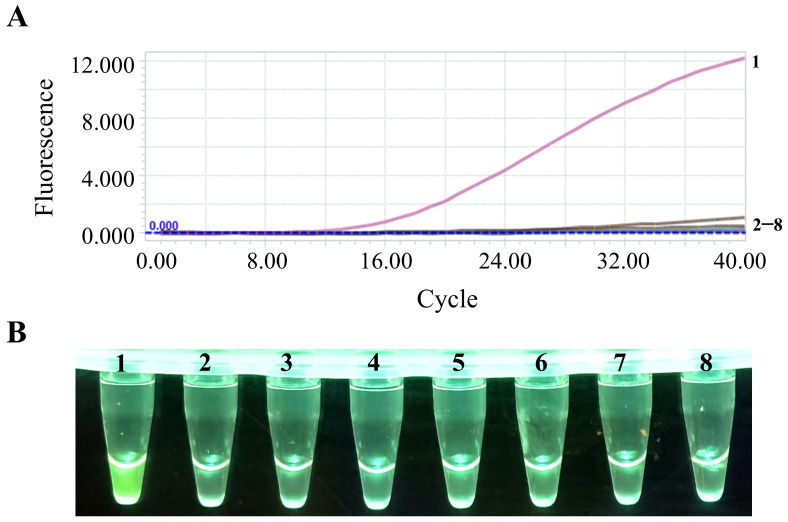
Specificity verification of the PEDV RT-RAA reaction system. (**A**) Real-time fluorescence detection results for specificity verification of the PEDV RT-RAA reaction system. Numbers 1–8 represent PEDV, PRRSV, CSFV, PRV, PCV2, PDCoV, PoRV, and TGEV, respectively. (**B**) Results of the blue-light detection for the specificity verification of the PEDV RT-RAA reaction system. Numbers 1–8 represent PEDV, PRRSV, CSFV, PRV, PCV2, PDCoV, PoRV, and TGEV, respectively.

Using the optimized PEDV liquid fluorescence RT-RAA reaction system, nucleic acids from PEDV, PRRSV, CSFV, PRV, PCV2, PDCoV, PoRV, and TGEV were amplified and detected. As shown in Figure 4, fluorescence signals were observed exclusively in PEDV-positive samples, while no fluorescence signals were detected in the other seven porcine viruses. These results demonstrate the high specificity of this method, with no cross-reactivity observed with other viruses.

### 3.4. Sensitivity Evaluation of the PEDV RT-RAA Reaction System

**Figure 5:**
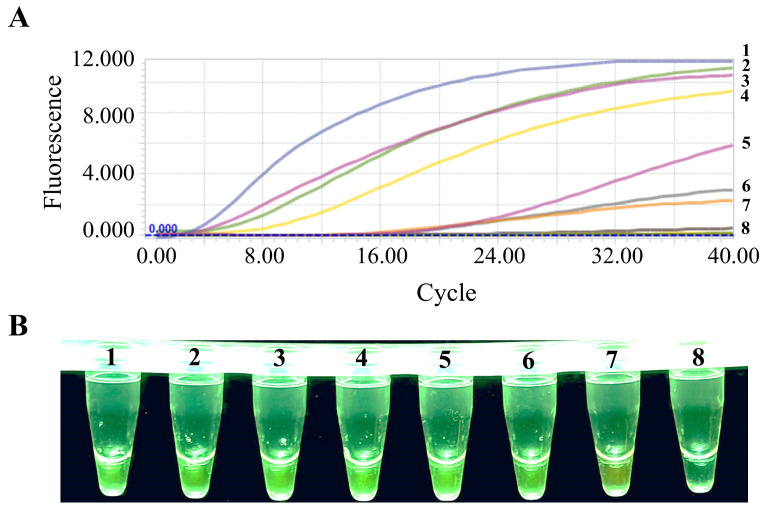
Sensitivity verification of the PEDV RT-RAA reaction system. (**A**) Real-time fluorescence detection results for sensitivity verification of the PEDV RT-RAA reaction system. Numbers 1–8 represent concentrations from 10^6^ to 1 copies/μL, as well as the negative control. (**B**) Results of the blue-light detection for the sensitivity verification of the PEDV RT-RAA reaction system. Numbers 1–8 represent concentrations from 10^6^ to 1 copies/μL, as well as the negative control.

The optimized PEDV RT-RAA reaction system was used to test the PEDV N gene standard plasmid ranging from 1 to 10^6^ copies/μL, with nuclease-free water as the negative control. Fluorescence signals were detectable at a template concentration as low as 1 copy/μL, as shown in Figure 5, highlighting the excellent sensitivity of this method.

### 3.5. Reproducibility Evaluation of the PEDV RT-RAA Reaction System

Using the optimized PEDV RT-RAA reaction system, positive standard plasmids at different dilution concentrations (10^5^, 10^4^, and 10^3^ copies/μL) were subjected to three replicate tests. Additional tests were performed on the second and third days to evaluate reproducibility. As shown in Table 2, fluorescence signals were measured using a real-time quantitative PCR instrument. The Ct values from the three replicates showed minimal variation, with inter-assay and intra-assay coefficients of variation both below 10%, indicating that this method exhibits high reproducibility.

### 3.6. Detection of Clinical Samples Using the PEDV RT-RAA Reaction System

To evaluate the performance of the PEDV RT-RAA reaction system, 63 clinical samples were tested using the established PEDV RT-RAA reaction system, and simultaneous testing was performed with RT-qPCR. As shown in Table 3, the method accurately distinguished between negative and positive samples, with 100% concordance with the RT-qPCR results, indicating that the PEDV RT-RAA reaction system established in this study has excellent detection performance.

## 4. Discussion

PEDV is a major pathogen of swine intestinal diseases, and its widespread transmission results in high morbidity and mortality rates among neonatal piglets, causing significant economic losses to the livestock industry [26]. Currently, the available PEDV vaccines offer insufficient immune protection, and no effective therapeutic measures are available [27]. To address this challenge, farms must implement preventive and control measures, including intervening at the source of virus transmission and strengthening epidemic monitoring, to effectively control the spread of PEDV. Timely monitoring of PEDV is crucial for controlling disease transmission. The symptoms of PEDV infection are clinically and pathologically indistinguishable from those caused by other diarrhea-inducing pathogens, such as TGEV, PoRV, and PDCoV [28,29,30], which makes diagnosis based solely on clinical signs and histopathology unreliable. Laboratory testing is essential for the differential diagnosis of PEDV and/or other diarrheal pathogens. Several laboratory diagnostic methods for PEDV have been reported, including IFA, IHC, ELISA, and RT-qPCR. Due to their rapid turnaround time and sensitivity, conventional and real-time RT-PCR systems are commonly employed for PEDV detection during epidemics, localized outbreaks, and for quarantine or slaughter policies [31]. However, these PCR methods require expensive instrumentation and complex procedures, which makes them unsuitable for resource-limited areas or outbreak sites. Thus, the development of a rapid, accurate, sensitive, and easy-to-use on-site detection method is crucial for preventing and controlling PED.

The rapid development of molecular biology techniques has led to the emergence of new molecular detection methods that are now widely applied. One such method is RAA, a novel isothermal amplification technique. Compared to traditional PCR, RAA does not require expensive equipment or consumables, has a broader application range, and is simpler to operate. RAA allows for the rapid amplification of DNA or RNA at lower temperatures, with amplification products obtained in just 30 min [25]. Compared to other isothermal amplification methods, such as LAMP, RAA is simpler, requiring only a pair of primers, and has a shorter runtime. In contrast, LAMP requires four to six primers, operates at 65 °C, and has a longer runtime [32]. Real-time fluorescence RAA combines the basic RAA system with a fluorescent probe system, enabling real-time monitoring of target gene amplification and meeting the needs for on-site detection. Real-time fluorescence RAA is currently widely used to detect pathogens such as viruses and bacteria [33,34,35]. In 2022, Zhang et al. developed an RT-RAA assay for the detection of PEDV. This assay only requires a 30 min isothermal reaction at 41 °C and provides strong specificity and high sensitivity. However, it still requires the use of a miniature fluorescence detector, which limits its use in remote areas where such equipment may not be available [36].

In this study, a rapid, convenient, and sensitive detection method for PEDV based on RT-RAA technology was developed, enabling on-site visual detection when combined with a blue light detection device. The core of establishing the RT-RAA detection method lies in the design of primers and probes, which requires a comprehensive analysis of the target sequence to create primers and probes with high amplification efficiency and strong specificity, while ensuring accuracy in practical applications. PEDV gene sequences were aligned to design specific primers and probes targeting the N gene, their performance was verified, and the optimal primers and probes for amplification were selected. The reaction system was further optimized, and its specificity, sensitivity, and stability were evaluated. The method exhibited no cross-reactivity with PRRSV, CSFV, PRV, PCV2, PDCoV, PoRV, or TGEV, demonstrating strong specificity. The minimum detection limit was 1 copy/μL, highlighting its exceptional sensitivity. When plasmids at high, medium, and low concentrations were subjected to three replicate tests, the inter-assay and intra-assay CV were all below 10%, confirming good reproducibility. To further validate the reliability of the system, 63 clinical samples (27 positive and 36 negative) were collected, and comparative tests were conducted using the PEDV RT-RAA reaction system and a commercial RT-qPCR kit. The results showed 100% concordance between the two methods. The RT-qPCR method takes approximately 60 min [37], whereas the RT-RAA method can be completed in just 20 min. When paired with commercial rapid nucleic acid extraction kits, the RT-RAA method can perform both nucleic acid extraction and detection within 30 min, providing a simpler operation without the need for large instruments. Additionally, compared to RT-qPCR, RT-RAA technology provides considerable cost advantages. The reagent cost of the PEDV RT-RAA reaction system is approximately $1.50, which is comparable to the cost of RT-PCR reagents. However, RT-qPCR requires expensive equipment, such as thermal cyclers, fluorescence detectors, and other specialized instruments, whereas RT-RAA only requires simple heating devices (such as small water baths or metal block heaters), making it better suited for point-of-care applications. In conclusion, the PEDV RT-RAA reaction system method developed in this study is sensitive, accurate, rapid, simple, and cost-effective, which makes it highly suitable for field applications and provides strong support for the early monitoring and precise control of PEDV.

## 5. Conclusions

This study developed a rapid detection method for PEDV based on RT-RAA technology, specifically designed for on-site PEDV detection. The method is portable, eliminates the need for large instruments, enables visual detection under blue light, and achieves high sensitivity and specificity. In clinical tests, it showed 100% concordance with RT-PCR, demonstrating its reliability and clinical potential. Future work will focus on validating its performance under diverse field conditions, particularly in resource-limited settings, and exploring its application to other swine pathogens. Overall, the PEDV RT-RAA reaction system provides a rapid, sensitive, and accurate tool for on-site detection of PEDV in pig farms.

## Figures and Tables

**Table 1:** PEDV primer and probe sequences.

Primer/Probe	Sequence (5′→3′)	Amplified Fragment (bp)
F	TCACAGAATCGTGGAAATAACCAGGGTCG	
R1	TGACAGCAGCCACCAGATCATCGCGTGATG	156
R2	CCCAAAGATTTAAGGGCATCCTTGACAGC	178
R3	TTTCTCCAATACCCAAAGATTTAAGGGCATC	189
P	AACAGAGGAGGCAATAATAATAACAATAACAAG/i6FAMdT//idSp//iBHQ1dT/CGTAACCAGTCCAAG/C3 Spacer/	

**Table 2:** Reproducibility verification of the PEDV RT-RAA reaction system.

Plasmid Concentration (Copies/μL)		Ct	Ct	Ct	CV (%)
Intra-assay	10^5^	3.01	3.25	3.12	3.84
	10^4^	9.00	8.42	8.26	4.55
	10^3^	12.30	12.37	13.33	4.54
Inter-assay	10^5^	3.42	3.82	3.32	7.52
	10^4^	9.76	9.63	9.11	3.62
	10^3^	13.71	13.03	15.69	9.77

**Table 3:** Clinical sample testing.

Test Method		RT-RAA			Coincidence Rate
		Positive	Negative	Total	
RT-qPCR	Positive	27	0	27	100%
	Negative	0	36	36	
	Total	27	36	63	

## Data Availability

The data are contained within the article.
